# Inclusion of Soluble Fiber During Gestation Regulates Gut Microbiota, Improves Bile Acid Homeostasis, and Enhances the Reproductive Performance of Sows

**DOI:** 10.3389/fvets.2021.756910

**Published:** 2021-11-17

**Authors:** Xiaoyu Wu, Shengnan Yin, Chuanshang Cheng, Chuanhui Xu, Jian Peng

**Affiliations:** ^1^Department of Animal Nutrition and Feed Science, College of Animal Science and Technology, Huazhong Agricultural University, Wuhan, China; ^2^The Cooperative Innovation Center for Sustainable Pig Production, Wuhan, China

**Keywords:** soluble fiber, gut microbiota, bile acid, reproductive performance, sow

## Abstract

Interaction between the dietary fiber and the gut microbes can regulate host bile acid metabolism. This study sought to explore the effects of guar gum combined with pregelatinized waxy maize starch (GCW) in a gestation diet on reproductive performance, gut microbiota composition, and bile acid homeostasis of sows. A total of 61 large white sows were randomly grouped into the control (*n* = 33) and 2% GCW (*n* = 28) groups during gestation. GCW diet increased birth-weight of piglets, and decreased the percentage of intrauterine growth restriction (IUGR) piglets. In addition, dietary GCW reduced gut microbial diversity and modulated gut microbial composition in sows on day 109 of gestation. The relative abundance of bile salt hydrolase (BSH) gene-encoding bacteria, *Lactobacillus* and *Bacteroides* decreased after GCW administration, whereas no significant difference was observed in the fecal level of total glycine-conjugated and taurine-conjugated bile acids between the two groups. Dietary GCW increased the relative abundance of *Ruminococcaceae* (one of few taxa comprising 7α-dehydroxylating bacteria), which was associated with elevated fecal deoxycholic acid (DCA) in the GCW group. GCW administration lowered the concentrations of plasma total bile acid (TBA) and 7α-hydroxy-4-cholesten-3-one (C4) (reflecting lower hepatic bile acid synthesis) at day 90 and day 109 of gestation compared with the control diet. Furthermore, the levels of plasma glycoursodeoxycholic acid (GUDCA), tauroursodeoxycholic acid (TUDCA) and glycohyocholic acid (GHCA) were lower in the GCW group compared with the control group. Spearman correlation analysis showed alterations in the composition of the gut microbiota by GCW treatment was associated with improved bile acid homeostasis and reproductive performance of sows. In conclusion, GCW-induced improves bile acid homeostasis during gestation which may enhance reproductive performance of sows.

## Introduction

Increased maternal total bile acid (TBA) of serum is implicated in dysregulation of bile acid homeostasis, which is associated with fetal distress (1), unexplained stillbirth (2), spontaneous preterm labor (3), neonatal low birth weight (4), and intrauterine fetal pig death (5). Notably, elevated maternal serum TBA levels are associated with accumulation of toxic bile acids (e.g., LCA) in placenta (6). In addition, bile acids can be transported across the placenta causing adverse fetal outcome in intrahepatic cholestasis of pregnancy (ICP) patients (7).

Synthesis and metabolism of bile acids is tightly regulated by gut microbiota (8). Alterations in gut microbiota composition and function can affect host bile acid metabolism (9, 10). Dietary fiber may serve as a platform for interaction between gut microbiota and bile acids and thus improve production of secondary bile acids (11). Secondary bile acids (e.g., glycoursodeoxycholic acid) influence host bile acids metabolism via the modulation of intestinal farnesoid X receptor (FXR) signaling (12). Diets high in whole grains increases the plasma levels of taurocholic acid (TCA) and glycocholic acid (GCA), which are implicated in activation of nuclear FXR target genes thus modulating bile acid metabolism (13). Soluble dietary fibers (such as wheat arabinoxylan and oat β-glucan) reduce the levels of circulating bile acids in swine model (14, 15). Notably, increase in serum bile acid levels is inhibited by oral guar gum treatment in women with ICP (16, 17). Our previous studies report that konjac flour supplementation during gestation alters gut microbiota composition in sows (18). Moreover, supplementing diet with 0.8% soluble dietary fiber can reduce incidence of intrauterine growth restriction (IUGR) piglets and improve intra-litter uniformity in replacement gilts (19). However, previous studies have not explored the mechanism by which soluble dietary fiber enhances reproductive performance in sows.

This study explored the impacts of dietary GCW intake on reproductive performance and gut microbiota composition and bile acid homeostasis of sows during gestation. The hypothesis of the study was that dietary GCW intake in gestation perturbs the gut microbiota accompanied with changes in bile acid composition in feces, and in turn induces intestinal FXR signaling, which may reduce TBA levels in plasma.

## Materials and Methods

Animal use and care protocol used in this study was approved by Institutional Animal Care and Use Committee of Huazhong Agricultural University. The ethical approval number of this study is HZAUSW-2016-023.

### Animals, Diets, and Sample Collection

GCW used in this experiment was obtained by mixing 85.7% pregelatinized waxy maize starch (Hangzhou, China) with 14.3% guar gum (Yunzhou, China). A total of 61 multiparous sows (large white, parity 2–6) were randomly grouped into two dietary treatments using a complete block design. During gestation, sows were fed with corresponding experimental diets: the control group was fed with basal diet (control, *n* = 33) whereas the GCW group was fed with basal diet supplemented with 2% GCW to replace rice bran meal (GCW, *n* = 28). All experimental diets were formulated to meet nutrient requirements of gestating sows according to recommendations by NRC (20). Treatments were isonitrogenous and isoenergetic diets. Diet compositions and nutrient levels are presented in Supplementary Table 1. Animals were fed twice a day (07:00 and 14:30 h). Blood samples (6~8 mL) were collected from the marginal ear vein of sow with disposable vacuum blood collection tubes 4 h after the morning meal (7~10 sows per group) on days 30, 60, 90, 109 of gestation (G30, G60, G90, and G109). Plasma was extracted by centrifugation of blood samples at 3,000 × g for 5 min at 4°C and stored at −80°C until analysis. Fresh feces were collected on day 109 of gestation and stored in a −80°C freezer. All sows did not present any signs of disease before sampling.

### Measurement of Plasma TBA, ALT, and AST

Plasma TBA, alanine aminotransferase (ALT), and alanine aminotransferase (AST), were determined through standard routine procedures using a Mindray BS-240 automatic biochemical analyzer (Mindray biomedical electronic co. LTD, Shenzhen, China).

### Measurement of Plasma FGF19 and C4

Concentrations of fibroblast growth factor 19 (FGF19) and 7α-hydroxy-4-cholesten-3-one (C4) in plasma were determined using porcine enzyme-linked immunosorbent assay kits (Nanjing Camilo biological engineering co. LTD, Nanjing, China), following the manufacturer's instructions.

### Quantification of *Baij, Bsh1*, and *Bsh2* Gene by Real-Time PCR

Total fecal microbial DNA was extracted using TIANamp Stool DNA Kit (Tiangen Biotech co. LTD, Beijing, China), following the manufacturer's instructions. Expression levels of bacterial bile acid metabolism-related genes (*baij, bsh1*, and *bsh2*) were determined by quantitative real-time PCR (Q-PCR). Expression levels of *baij, bsh1*, and *bsh2* genes were normalized using 16s rDNA. The reaction system and conditions used for Q-PCR were as described in a previous study (21). Primer sequences used in this study are presented in Supplementary Table 2.

### Bile Acid Analysis

All bile acid standards were purchased from Olchemim Ltd. (Olomouc, Czech Republic) and Sigma (St. Louis, MO, USA). For plasma samples, 200 μL methanol was added to 50 μL plasma-spiked with 10 μL internal standard (IS), vortexed, and centrifuged at 12,000 r/min for 10 min. For feces samples, 20 mg of feces were spiked with 10 μL IS and mixed with 200 μL methanol, vortexed, and centrifuged at 12,000 r/min for 10 min. The supernatant were evaporated to dryness, reconstituted in 100 μL 50% aqueous methanol (V/V) for further LC-MS analysis.

Bile acids were quantified using ultra-performance liquid chromatography (UPLC, Shim-pack UFLC SHIMADZU CBM30A system) coupled to a tandem mass spectrometry system (MS/MS, Applied Biosystems 6500 QTRAP). Chromatographic separations were performed with an ACQUITY UPLC HSS C18 column (1.8 μm, 2.1 mm 100 mm, Waters Corp., Milford, MA, USA). The mobile phase was water (1,000 mL) + formicacid (100 μL) + ammonium acetate (5 mM; A) and formicacid (100 μL) + acetonitrile (1,000 mL; B). The gradient conditions were as follows: 0–0.5 min (5% B), 0.5–4.5 min (40–50% B), 4.5–7.5 min (50–80% B), 7.5–10 min (80–95% B), and 10–12 min (5% B). The total analysis time was 12 min and the injection volume was 3 μL. The column temperature was maintained at 40°C with the flow rate set at 0.35 mL/min. The mass spectrometer was operated with an electrospray ionization interface in negative ionization (ESI–) mode. The UPLC-MS raw data were collected using Multiple Reaction Monitoring (MRM) mode. The qualitative analysis of mass spectrometry data was performed based on the self-built database MWDB (Metware Biotechnology Co., Ltd., Wuhan, China), and the quantitative analysis were performed using the Multiquant 3.0 software (Sciex, Framingham, MA, USA).

### Analysis of Gut Microbiota

Total bacterial DNA was extracted from fecal samples using a QIAamp DNA Stool Mini Kit (Qiagen, Germany) following the manufacturer's protocol. Bacterial 16S rRNA gene V3-V4 region of each sample was then amplified using 16S rRNA universal primers. PCR products were mixed then sequenced on an Illumina MiSeq platform (2 × 250 bp) (Illumina, United States). Microbiome bioinformatics were performed with QIIME2 2019.4 (22) with slight modification according to the official tutorials. Briefly, raw sequence data were demultiplexed using the demux plugin following by primers cutting with cutadapt plugin (23). Sequences were then quality filtered, denoised, merged, and chimera removed using the DADA2 plugin (24). Non-singleton amplicon sequence variants (ASVs) were aligned with mafft (25) and used to construct a phylogeny with fasttree2 (26). Alpha-diversity values of the samples were determined based on the Observed species, Chao1, Shannon, and Simpson index. Beta-diversity measures were calculated using weighted-UniFrac distance. Linear discriminant analysis (LDA) effect size analysis (LEfSe) was used to identify key gut microbiota of the two groups (*p* < 0.05, Wilcoxon rank-sum test; log LDA > 2).

### Statistical Analysis

Data were analyzed with SPSS (SPSS 23.0, SPSS Inc., Chicago, IL). Categorical variables were analyzed using χ^2^-tests. Normally distributed data were analyzed using student's unpaired *t*-test; whereas data that was not normally distributed was analyzed using non-parametric Mann Whitney *U*-test. Spearman correlation analyses were performed using Prism GraphPad 8.0.1 (GraphPad Software, San Diego, CA, United States). *P* < 0.05 were considered significant.

## Results

### Reproductive Performance

Effects of GCW in the gestation diet on reproductive performance of sows are presented in Table 1. GCW showed no significant effect on total number of born piglets and number of alive piglets. GCW significantly increased birth weight of piglets compared with the weight of piglets in the control group. Moreover, the percentage of IUGR piglets (< 800 g) in the total alive piglets for the GCW group was 0.91%, which was significantly lower compared with that of the control group (3.89%). However, no significant differences were observed for the number of piglets with birth weights ranging between 800–1,000, 1,000–1,200, 1,200–1,400, and more than 1,400 g between the two groups.

**Table 1:** Effects of dietary supplementation with GCW during gestation on reproductive performance of sows^a^.

**Parameter**	**CON**	**GCW**	***P*-value**
No. of litters	33	28	
**Litter size, No/litter**			
Total born	12.97 ± 2.59	12.07 ± 2.65	0.187
Born alive	12.45 ± 2.53	11.82 ± 2.56	0.337
Birth weight of litters, kg	18.36 ± 3.91	18.04 ± 3.61	0.747
Birth weight of piglets, kg	1.47 ± 0.33	1.53 ± 0.32	0.030
**Birth weight distribution of piglets, %** ^**b**^			
<800 g, %	3.89 (16)	0.91 (3)	0.010
800–1,000 g, %	4.62 (19)	5.13 (17)	0.746
1,000–1,200 g, %	8.76 (36)	10.88 (36)	0.333
1,200–1,400 g, %	18.98 (78)	19.64 (65)	0.821
>1,400 g, %	63.75 (262)	63.44 (210)	0.932

a *Data on fetus and birth weight distribution of piglets are expressed as proportion of total born piglets in both groups (actual number in the group); other data are presented as mean ± SD*.

b *The birth weight distribution of piglets refers to the ratio of piglets in each weight range to the total live-born piglets in both groups*.

### Gut Microbiota Analysis

**Figure 1:**
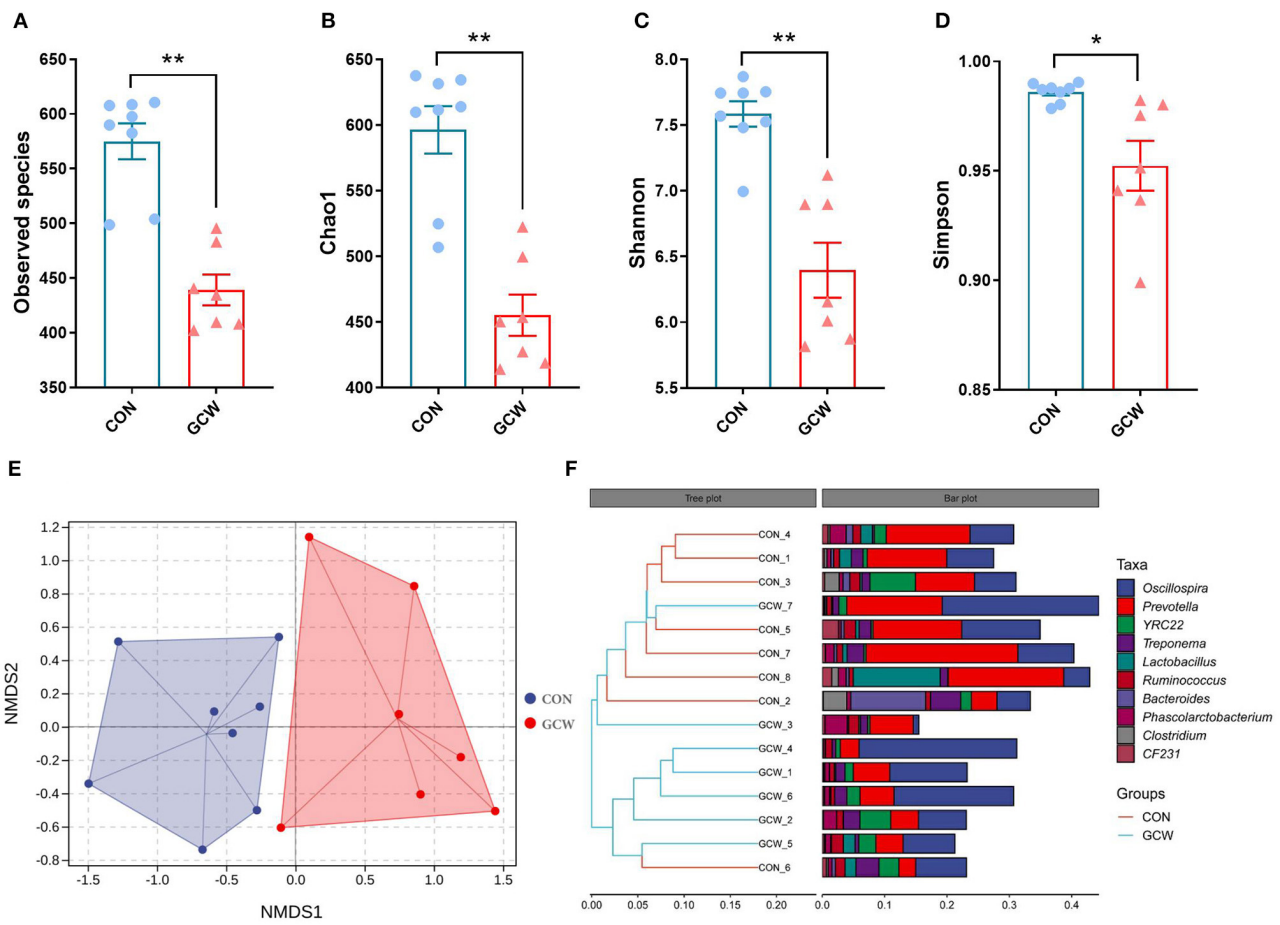
Alpha and beta diversity of gut microbiota. The number of Observed species **(A)**, Chao 1 **(B)**, Shannon **(C)**, and Simpson index **(D)** between control (*n* = 8) and GCW (*n* = 7) groups. **(E)** Non-metric multidimensional scaling (NMDS) plot. **(F)** Dendrogram of hierarchical clustering analysis. Data are presented as means ± SEM. ^*^p < 0.05, ^**^p < 0.01.

To explore the composition of gut microbiota for the two groups on day 109 of gestation, 16S ribosomal RNA gene (V3-V4 gene regions) sequencing was conducted. Analysis of Alpha-diversity indexes showed that the number of observed species, Chao1, Shannon, and Simpson index were significantly lower in the GCW group compared with the control group (Figures 1A–D). Nonmetric multidimensional scaling (NMDS) analysis for beta density showed that the control group was separately clustered from the GCW group (Figure 1E). Hierarchical Cluster Analysis (HCA) based on the weighted-UniFrac distance showed that control samples clustered together whereas GCW samples clustered together (Figure 1F). This finding validated the results obtained by NMDS analysis.

**Figure 2:**
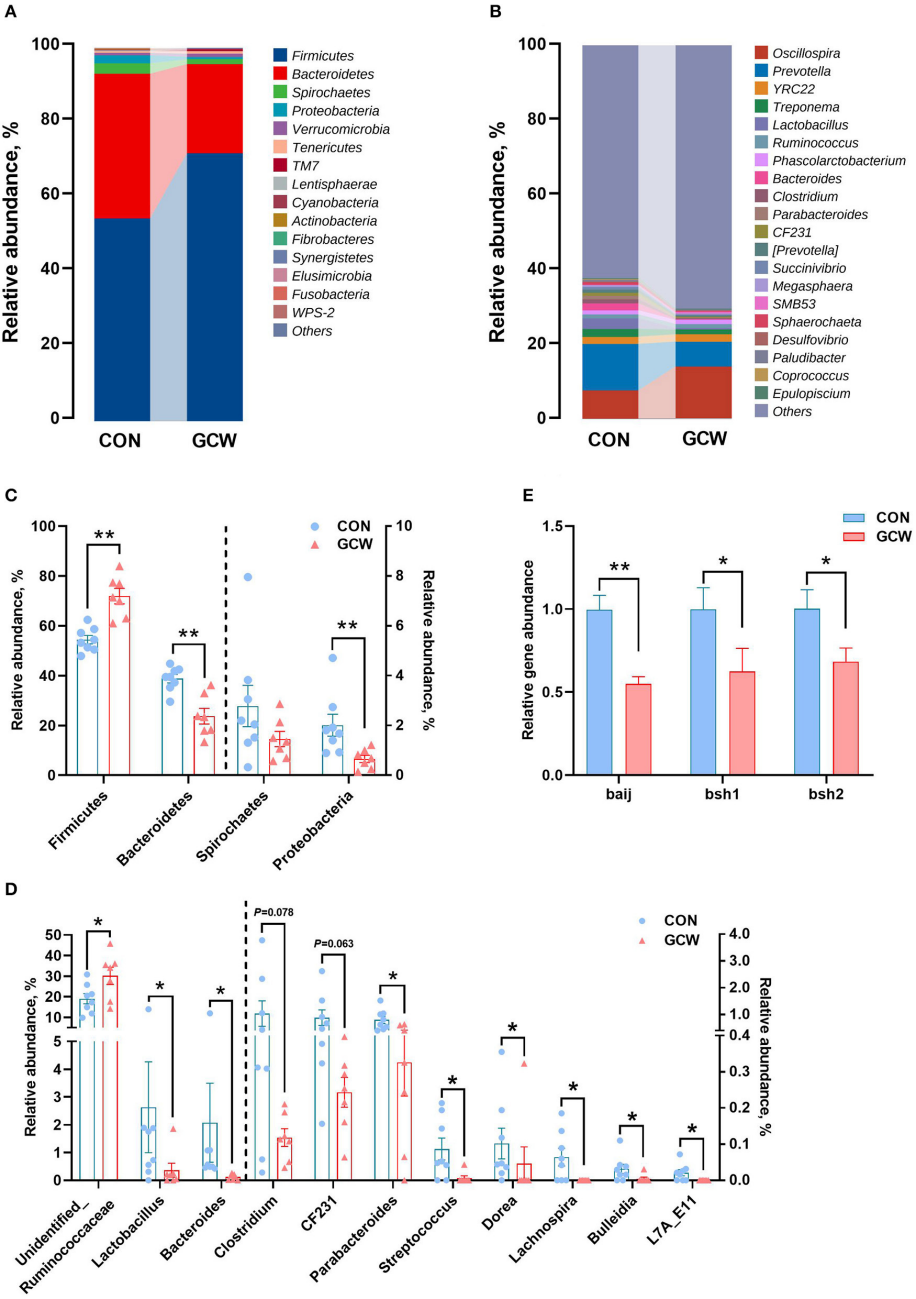
Gut microbiota composition profiles. Bar plot showing relative abundance of microbiota at the phylum **(A)** and genus **(B)** levels for the control and GCW groups. Different levels of bacteria at the phylum **(C)** and genus **(D)** levels between control (*n* = 8) and GCW (*n* = 7) groups. **(E)** Relative abundance of genes involved in bile acid 7α-dehydroxylation (*baij*) and bile salt hydrolysis (*bsh1* and *bsh2*) between control (*n* = 15) and GCW (*n* = 14) groups. Data are presented as means ± SEM. ^*^p < 0.05, ^**^p < 0.01.

*Firmicutes, Bacteroidetes, Spirochaetes*, and *Proteobacteria* were the four most dominant phyla (Figure 2A). At the genus level, *Oscillospira, Prevotella, YRC22*, and *Treponema* were the four most abundant genera (Figure 2B). The GCW group showed higher abundances of *Firmicutes* and lower abundances of *Bacteroidetes* and *Proteobacteria* compared with the control group (Figure 2C). The GCW group had lower abundances of *Lactobacillus, Bacteroides, Parabacteroides, Streptococcus, Dorea, Lachnospira, Bulleidia*, and *L7A_E11* and greater abundances of *Unidentified_Ruminococcaceae* compared with the control group (Figure 2D). In addition, GCW treatment tended to reduce the relative abundance of *Clostridium* and *CF231* compared with the levels in the control group (Figure 2D). At the family level, the GCW group showed higher abundances of *Ruminococcaceae* and lower abundances of *Lactobacillaceae, Bacteroidaceae*, and *RF16* compared with the levels in the control group (Supplementary Figure 1). Notably, dietary GCW supplementation significantly reduced expression level of *baij* gene for *Clostridium scinden, bsh1* gene for *Lactobacillus plantarum*, and *bsh2* gene for *Bacteroides ovatus* compared with the levels in the control group (Figure 2E). LEfSe analysis showed similar changes in composition of gut microbiota at the phyla, family, and genus taxonomic levels (Supplementary Figure 2).

### Bile Acid Homeostasis-Related Parameters in Plasma

**Figure 3:**
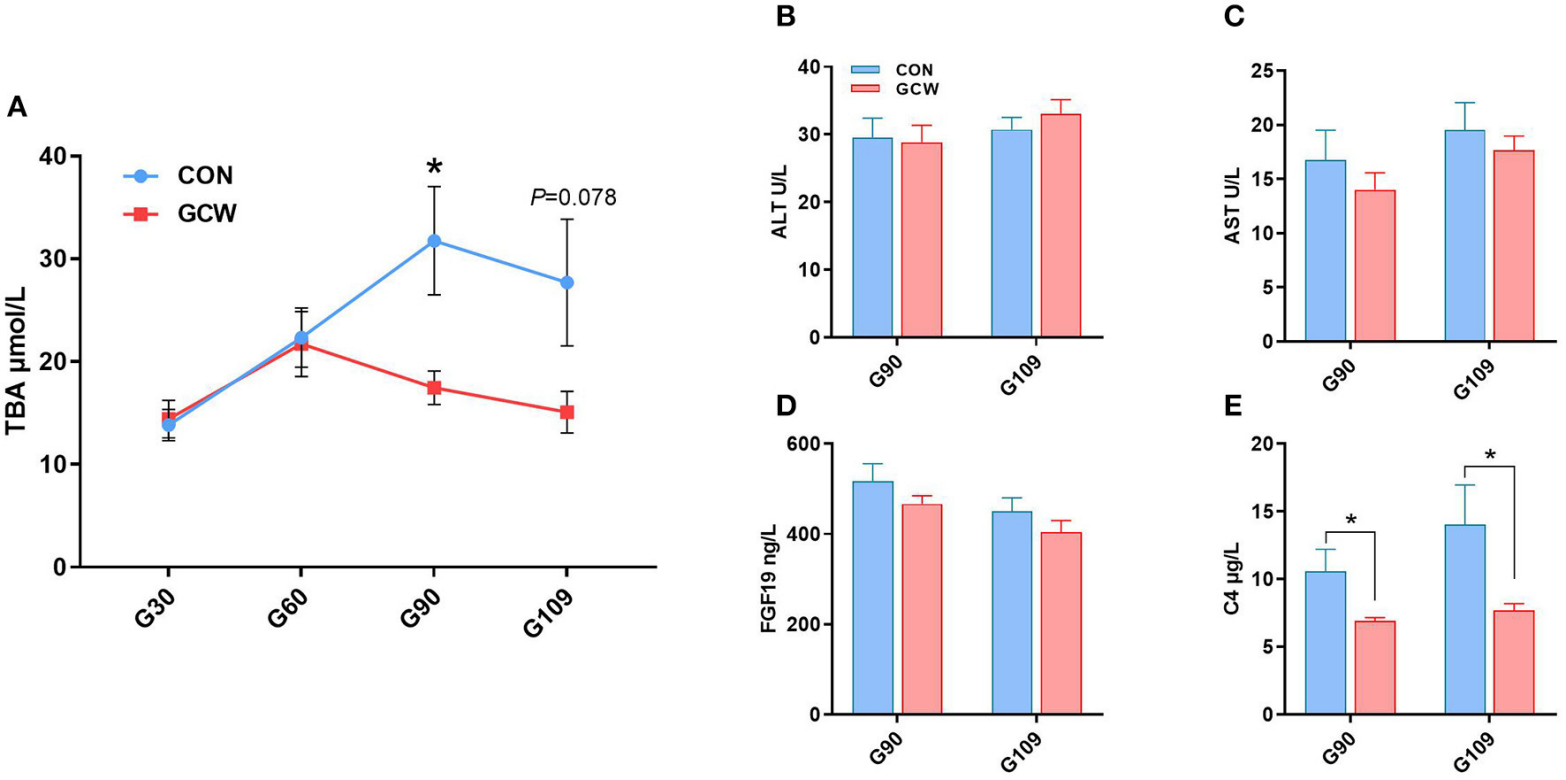
**(A)** Dynamic change of maternal peripheral plasma TBA between the two groups across gestation (*n* = 7–10/group). Plasma concentrations of ALT **(B)**, AST **(C)**, FGF19 **(D)**, and C4 **(E)** between the two groups during late gestation (*n* = 8–10/group). Data are presented as means ± SEM. ^*^p < 0.05.

To explore the effect of GCW on bile acid homeostasis, levels of several related plasma biological parameters were determined. Plasma TBA levels in the GCW group on day 90 of gestation were significantly reduced by 45.04% compared with the levels in the control group (Figure 3A). Moreover, GCW treatment tended to decrease plasma TBA level on day 109 of gestation compared with the level in the control group (Figure 3A). However, the two groups showed no significant difference in the levels of plasma ALT, AST, and FGF19 during late gestation (days 90 and 109) (Figures 3B–D). Notably, GCW diet significantly reduced plasma C4 concentration during late gestation compared with the control diet (Figure 3E).

### Plasma Bile Acids Composition

**Figure 4:**
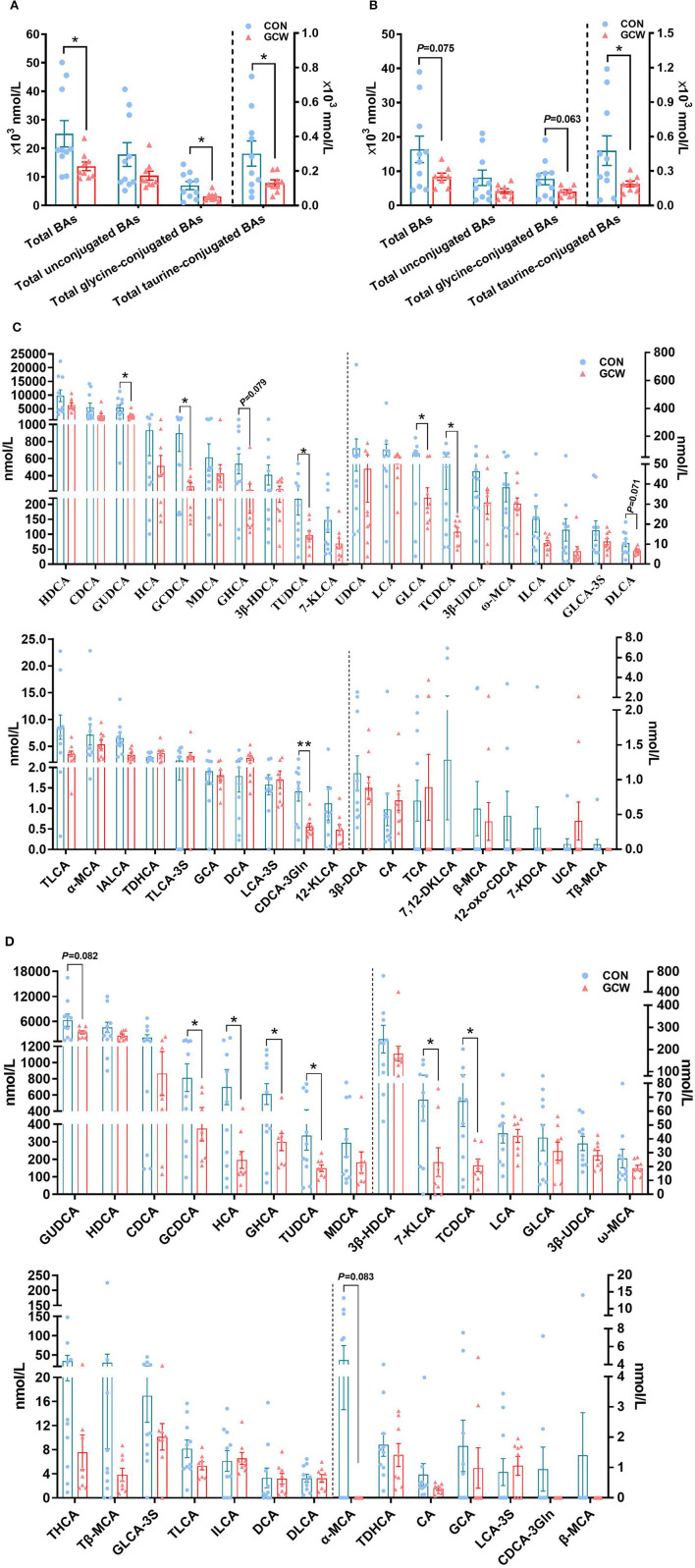
Plasma bile acid composition between the two groups at day 90 and day 109 of gestation. Plasma total bile acids, total unconjugated bile acids, total glycine-conjugated, and total taurine-conjugated bile acids at day 90 **(A)** and day 109 **(B)** of gestation. Plasma individual bile acids at day 90 **(C)** and day 109 **(D)** of gestation. Data are presented as means ± SEM (*n* = 8–10/group). ^*^p < 0.05, ^**^p < 0.01.

To further characterize the effect of GCW feeding on bile acid homeostasis of sows, we analyzed bile acids composition in plasma samples obtained from control and GCW groups. Most plasma bile acids were unconjugated, followed by glycine-conjugated bile acids and taurine-conjugated bile acids (Figures 4A,B). Furthermore, analysis of individual bile acids showed high abundance of HDCA and CDCA and GUDCA bile acids in plasma (Figures 4C,D). In addition, the concentrations of plasma total bile acids, total glycine-conjugated bile acids and total taurine-conjugated bile acids in the GCW group on day 90 of gestation were significantly lower compared with the levels in the control group (Figure 4A). Moreover, concentrations of total taurine-conjugated bile acids were significantly reduced by GCW treatment, and level of total bile acids and total glycine-conjugated bile acids tended to decrease on day 109 of gestation in the GCW group compared with the control group (Figure 4B). Plasma levels of GUDCA, GCDCA, TUDCA, GLCA, TCDCA, and CDCA-3Gln were significantly decreased in GCW group on day 90 of gestation compared with the control group (Figure 4C). In addition, GCW intervention induced lower levels of GCDCA, HCA, GHCA, 7-KLCA, and TCDCA in plasma of sows on day 109 of gestation (Figure 4D).

### Fecal Bile Acids Composition

**Figure 5:**
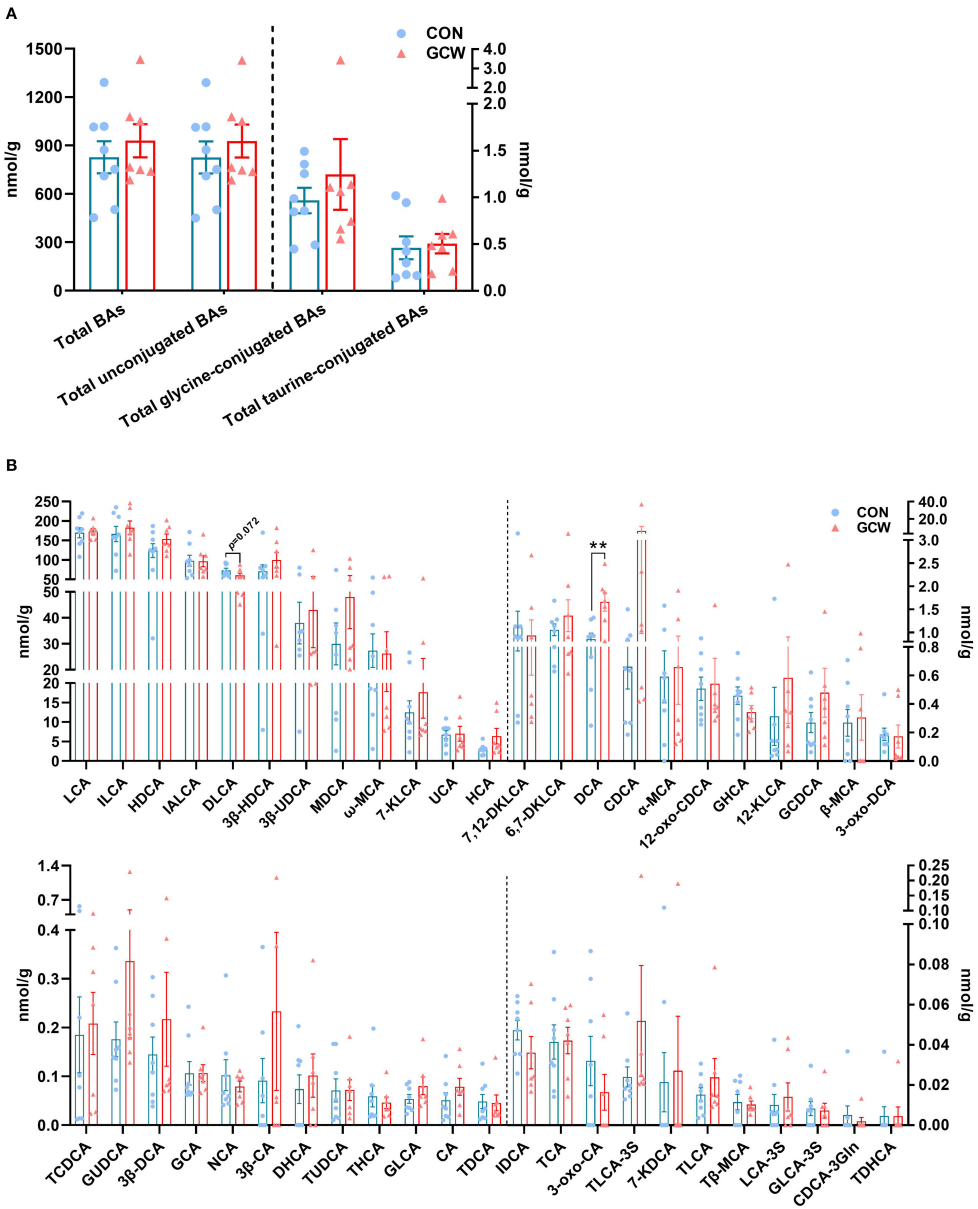
Fecal bile acid composition between the two groups on day 109 of gestation. **(A)** Fecal total bile acids, total unconjugated bile acids, total glycine-conjugated bile acids, and total taurine-conjugated bile acids. **(B)** Fecal individual bile acids. Data are presented as means ± SEM (*n* = 8 sows in the control group and 7 sows in the GCW group). ^**^p < 0.01.

Alterations in gut microbial flora induced by GCW treatment affected fecal bile acids composition. Predominant bile acids in fecal samples were unconjugated, followed by glycine-conjugated bile acids and taurine-conjugated bile acids (Figure 5A). Notably, LCA and ILCA and HDCA and IALCA were the most abundant fecal bile acids (Figure 5B). GCW had no significant effect on fecal total bile acids, total unconjugated bile acids, total glycine-conjugated bile acids or total taurine-conjugated bile acids (Figure 5A). In addition, GCW treatment upregulated the levels of fecal HDCA, 3β-HDCA, MDCA, 7-KLCA, HCA, DCA, and CDCA, with higher levels observed for DCA (Figure 5B). However, the levels of fecal DLCA decreased in GCW group compared with the control (Figure 5B).

### Correlation Between Microbiome and Parameters of Bile Acid Metabolism and Reproductive Performance of Sows

**Figure 6:**
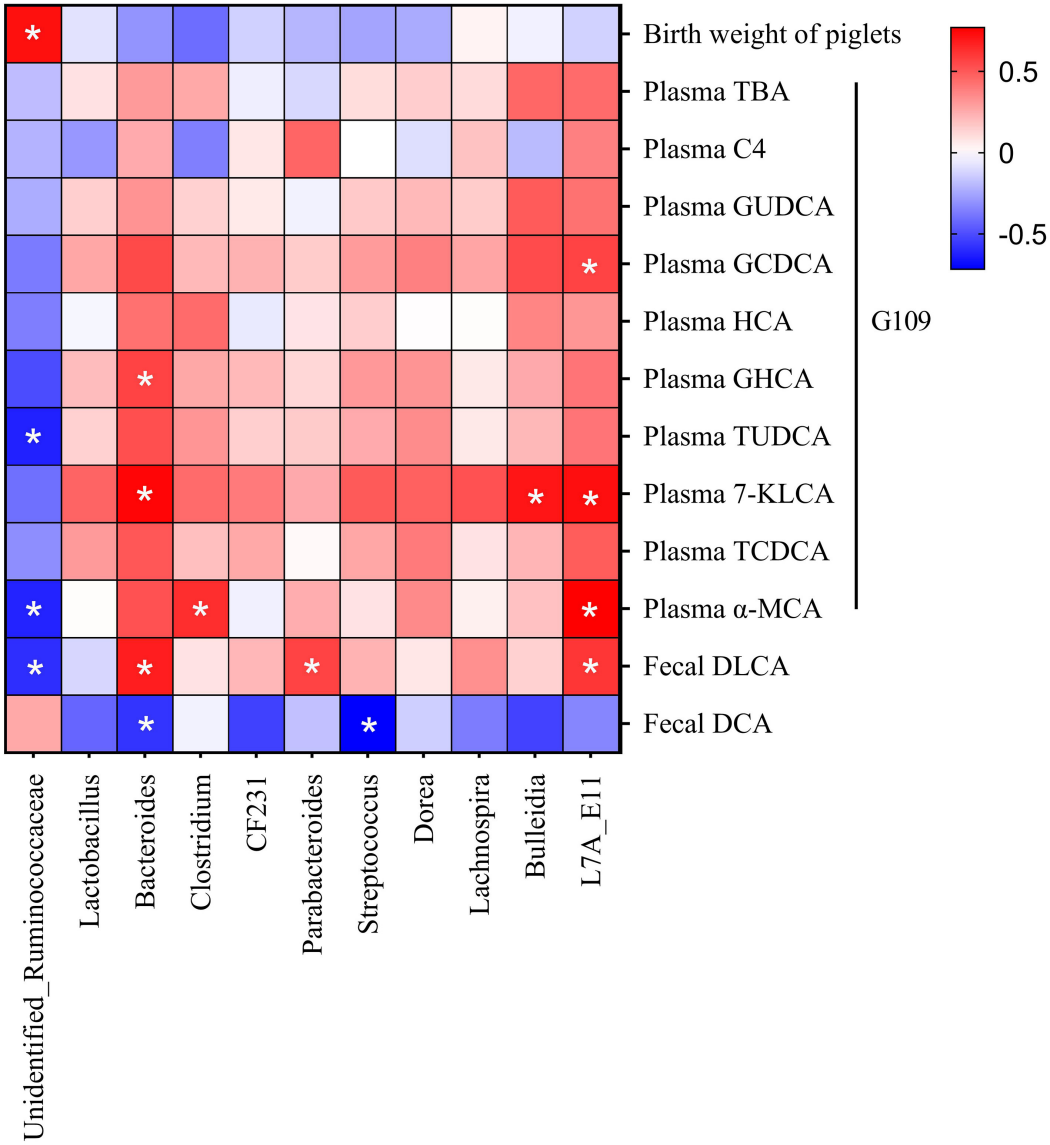
Correlation between microbiome and parameters of bile acid metabolism, and reproductive performance of sows. Red indicates positive correction, and blue indicates negative correction. ^*^p < 0.05.

Spearman correlation analysis was conducted to explore the potential link between microbiome and parameters of bile acid metabolism and reproductive performance of sows (Figure 6). *Unidentified_Ruminococcaceae* was positively correlated with birth weight of piglets, meanwhile negatively correlated with plasma TUDCA, plasma α-MCA, and fecal DLCA. *Bacteroides* was negatively correlated with fecal DCA, but positively correlated with plasma GHCA, plasma 7-KLCA and fecal DLCA. Additionally, *Clostridium* was positively correlated with plasma α-MCA, Parabacteroides was positively correlated with fecal DLCA, and *Streptococcus* was negatively correlated with fecal DCA.

## Discussion

Previous studies have explored interaction between dietary fiber, bile acids, and the gut microbiota (11). However, the effects of dietary soluble fiber on bile acid homeostasis during gestation have not been fully explored. Bile acids bind to the nuclear receptor FXR, which is primarily expressed in hepatocytes and distal ileal enterocytes, and thus regulate their own biosynthesis and transport (27). A recent study on pregnant women and mice reported reduced ileal FXR-mediated suppression of bile acid synthesis in the liver, resulting in hypercholanemia (28). In this study, dietary GCW treatment reduced plasma C4 level (reflecting lower hepatic bile acid synthesis). GCW-fed sows showed lower plasma primary bile acids, such as GCDCA, GHCA, and TCDCA, implying that GCW reduced hepatic bile acid synthesis. Bile acids such as HCA species (e.g., HCA and HDCA), GUDCA and TUDCA are antagonists of FXR (12, 29). Furthermore, plasma levels of GUDCA, TUDCA and GHCA were lower in the GCW group, which inhibited FXR signaling activity thus increasing hepatic bile acid synthesis. Notably, the levels of plasma conjugated bile acids were lower in the GCW group than in controls, which implies that conjugated bile acids were harmful to fetal pigs.

Guar gum binds to bile acids in the intestinal lumen and enhances their excretion in feces (30), which, in turn, lowers TBA concentration in plasma. However, concentration of fecal total bile acids was not significantly different between the two groups, which could be due to differences in fiber composition. The fecal total bile acid excretion was not evaluated in this, which is a potential limitation to our study. An increased intestinal permeability was detected in ICP patients during pregnancy, which may enhance the absorption of bacterial endotoxin (31). Furthermore, the disorder of bile acid metabolism in the late stage of pregnant mice could be induced by intraperitoneal injection of lipo-polysaccharide (LPS) (32). Notably, dietary GCW treatment reduced intestinal permeability and plasma endotoxin level (date not shown) and increased relative abundances of butyrate-producing bacteria (*Ruminococcaceae*) on day 109 of gestation. Also, GCW-fed sows showed higher plasma and fecal butyrate levels on day 109 of gestation (date not shown), which led to a strengthened gut barrier function. Therefore, the present study did not exclude that dietary GCW treatment reduced plasma TBA levels via downregulating plasma endotoxin levels of sows during gestation.

Previous studies report that dietary fiber modulates intestinal microbiota composition (33). Decreased microbiome diversity is a feature of dysbiosis (34). In this study, dietary GCW treatment reduced microbiome alpha diversity, possibly by selectively inhibiting the growth of harmful microorganisms (such as several members of the phyla *Proteobacteria*), which was consistent with findings from prior studies (35, 36). In the large intestine, gut microbiota-derived bile salt hydrolases (BSH) deconjugates host-derived conjugated primary bile acids to produce free primary bile acids, taurine and glycine (9). Metagenomic analyses show presence of BSH genes in Gram-positive bacteria genera (e.g., *Lactobacillus, Bifidobacterium, Enterococcus, Clostridium*, and *Listeria*) and Gram-negative bacteria genera (e.g., *Bacteroides*) (37). Upon deconjugation, the hydroxyl group at the C7 position of bile acids is then removed by 7α-dehydroxylating bacteria (e.g., *Clostridium* and *Eubacterium*), thus converting primary bile acids to secondary bile acids (38). In this study, dietary soluble fiber treatment increased the relative abundance of *Ruminococcaceae*, which was correlated with increase in the level of fecal DCA. Notably, members of *Ruminococcaceae* are can carry out 7α-dehydroxylation resulting in formation of secondary bile acids (39, 40). The *bsh1* and *bsh2* genes participated in converting conjugated bile acids to unconjugated bile acids (41). The *baij* gene could participate in the process of converting primary bile acids into secondary bile acids (42). Microbiota-derived bile acids, LCA and DCA and their taurine and glycine conjugated forms have high affinities for G protein Bile acid-activated receptor (GPBAR)-1, also known as TGR5 (43). Furthermore, the relative abundance of BSH-encoding bacteria (including *Lactobacillus* and *Bacteroides*) were lower in the GCW group compared with the levels in the control group. However, the concentration of fecal total glycine-conjugated and taurine-conjugated bile acids were not significantly different between two groups. Notably, the abundance of bile acid-metabolizing intestinal bacteria coincided with the expression levels of bacterial bile acid metabolism-related genes.

## Conclusion

The findings of this study show that dietary GCW intervention can enhance reproductive performance probably by maintaining bile acid homeostasis in sows during gestation. Moreover, dietary GCW treatment increased levels of fecal DCA by regulating gut microbiota in sows.

## Data Availability Statement

The datasets presented in this study can be found in online repositories. The names of the repository/repositories and accession number(s) can be found at: https://www.ncbi.nlm.nih.gov/bioproject/PRJNA771933.

## Ethics Statement

The animal study was reviewed and approved by Institutional Animal Care and Use Committee of Huazhong Agricultural University. Written informed consent was obtained from the owners for the participation of their animals in this study.

## Author Contributions

JP conceived and designed the experiments and wrote and revised the manuscript. XW performed the experiments, analyzed the data, and wrote part of the manuscript. SY and CC performed the experiments and took part in the data analysis. CX analyzed the data. All authors contributed to the article and approved the submitted version.

## Funding

This research was supported by the National Natural Science Foundation of China (31772609) and Hubei Province Science and Technology Innovation Major Project (2019ABA081).

## Conflict of Interest

The authors declare that the research was conducted in the absence of any commercial or financial relationships that could be construed as a potential conflict of interest.

## Publisher's Note

All claims expressed in this article are solely those of the authors and do not necessarily represent those of their affiliated organizations, or those of the publisher, the editors and the reviewers. Any product that may be evaluated in this article, or claim that may be made by its manufacturer, is not guaranteed or endorsed by the publisher.

## Abbreviations

TBA: total bile acid
FGF19: fibroblast growth factor 19
C4: 7α-hydroxy-4-cholesten-3-one
ALT: alanine aminotransferas
AST: alanine aminotransferase
FXR: farnesoid X receptor
TGR5: Takeda G protein-coupled receptor
TNF-α: tumor necrosis factor-α
BSH: bile salt hydrolase
LC-MS/MS: liquid chromatography tandem mass spectrometry
12-KLCA: 12-ketolithocholic acid
12-oxo-CDCA: 12-oxochenodeoxycholic acid
3-oxo-CA: 3-oxocholic acid
3-oxo-DCA: 3-oxodeoxycholic acid
3β-CA: 3β-cholic acid
3β-DCA: 3β-deoxycholic acid
3β-HDCA: β-hyodeoxycholic acid
3β-UDCA: 3β-ursodeoxycholic acid
6, 7-DKLCA, 6: 7-diketolithocholic acid
7, 12-DKLCA, 7: 12-diketolithocholic acid
7-KDCA: 7-ketodeoxycholic acid
7-KLCA: 7-ketolithocholic acid
CA: cholic acid
CDCA-3Gln: chenodeoxycholic acid-3-β-D-glucuronide
CDCA: chenodeoxycholic acid
DCA: deoxycholic acid
DHCA: dehydrocholic acid
DLCA: dehydrolithocholic acid
GCA: glycocholic acid
GCDCA: glycochenodeoxycholic acid
GHCA: glycohyocholic acid
GLCA: glycolithocholic acid
GLCA-3S: glycolithocholic acid-3-sulfate
GUDCA: glycoursodeoxycholic acid
HCA: hyocholic acid
HDCA: hyodeoxycholic acid
IALCA: isoallolithocholic acid
IDCA: isodeoxycholic acid
ILCA: isolithocholic acid
LCA: lithocholic acid
LCA-3S: lithocholic acid-3-sulfate
MDCA: murideoxycholic acid
NCA: norcholic acid
TCA: Taurocholic acid
TCDCA: taurochenodeoxycholic acid
TDCA: taurodeoxycholic acid
TDHCA: taurodehydrocholic acid
THCA: taurohyocholic acid
TLCA: taurolithocholic acid
TLCA-3S: taurolithocholic acid-3-sulfate
TUDCA: tauroursodeoxycholic acid
Tβ-MCA: tauro-β-muricholic acid
UCA: ursocholic acid
α-MCA: α-muricholic acid
β-MCA: β-muricholic acid
ω-MCA: ω-muricholic acid.
