# Supplementary material for: Inclusion of Soluble Fiber During Gestation Regulates Gut Microbiota, Improves Bile Acid Homeostasis, and Enhances the Reproductive Performance of Sows
Source: Front Vet Sci. 2021 Nov 17;8:756910. doi: 10.3389/fvets.2021.756910 (PMC8635514; doi:10.3389/fvets.2021.756910)
Supplement: Supplementary file 1 [file Data_Sheet_1.pdf]

Supplementary Table S1. Ingredients and nutrient composition of experimental gestation diets (as-fed basis).

Items	CON	GCW
Ingredient, %		
Corn	54.80	54.80
Soybean meal, 43%CP	11.50	11.50
Wheat bran	0.00	0.00
Soybean hull	14.00	14.00
Rice bran meal	16.00	14.00
Calcium carbonate	1.20	1.20
Dicalcium phosphate	1.00	1.00
Salt	0.40	0.40
Mildewcide[2]	0.10	0.10
Choline chloride	0.00	0.00
Fiber source	0.00	2.00
Premix[3]	1.00	1.00
Nutrient composition		
Net energy, MJ/kg[4]	9.53	9.53
Crude protein, %	13.96	13.81
ISF, %[5]	26.95	26.78
GCW, %[6]	2.85	4.04
NDF, %[7]	20.29	20.21
ADF, %[8]	5.47	5.42
Lys, %[4]	0.59	0.59
Ca, %[4]	0.78	0.75
Available P, %[4]	0.40	0.40

[1]GCW = 2% fiber of pregelatinized waxy maize starch (85.7%) plus guar gum (14.3%).
[2]Mildewcide: ammonium propionate.
[3]Provided per kg of diet: Cu, 30 mg; Fe, 160 mg; Zn, 160 mg; Mn, 55 mg; I, 0.5 mg; Se, 0.5 mg; Co, 0.8 mg; Cr, 0.2 mg; Vitamin A, 14000 IU; Vitamin D_3, 2900 IU; Vitamin E, 120 mg; Vitamin K_3, 6 mg; Vitamin B_1, 2.4 mg; Vitamin B_2, 8.5 mg; Vitamin B_6, 4.5 mg; Vitamin B_{12}, 0.03 mg; Vitamin H, 0.55 mg; Pantothenic acid, 30 mg; Folic acid, 5 mg; Nicotinamide, 50 mg.
[4]Calculated chemical concentrations using values for feed ingredients from the NRC (2012).
[5]insoluble fiber.
[6]solubel fiber.
[7]neutral detergent fiber.
[8]acid detergent fiber.

Supplementary Table S2. Primers used for real-time PCR analysis in this study.

Target gene	Forward primer (5`-3')	Reverse primer (5'-3')	Annealing temp.(°C)	Reference
Total bacteria	GTGSTGCAYGGYYGTCGTCA	ACGTCRTCCMCNCCTTCCTC	60	Maeda *et al*., 2003
baiJ	TCAGGACGTGGAGGCGATCCA	TACRTGATACTGGTAGCTCCA	60	Yoshimoto *et al*., 2013
bsh1	ATGGGCGGACTAGGATTACC	TGCCACTCTCTGTCTGCATC	54	Duary *et al*., 2012
bsh2	AATCGGCTCATCTGCACCAA	GTGGTCACGCAGGATAGAGG	60	This study

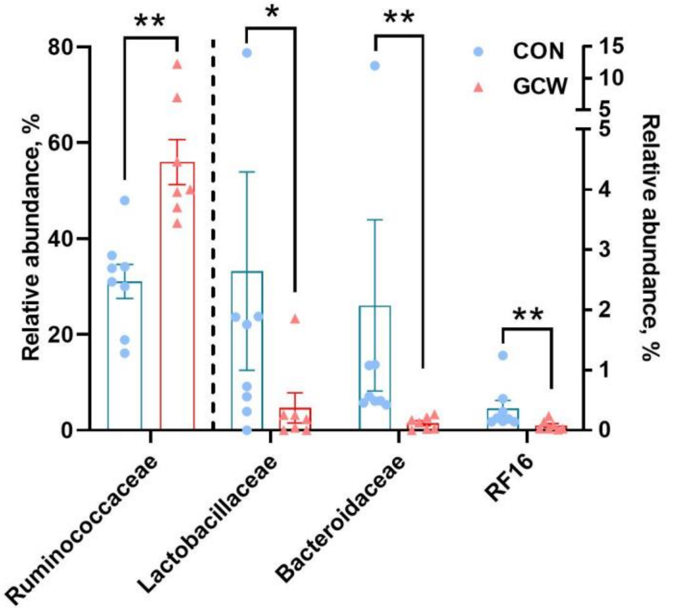

Supplementary Figure. S1. Significantly changed bacteria at family level between control (n = 8) and GCW (n = 7) groups. Data are shown as means ± SEM. *$p < 0.05$, **$p < 0.01$.

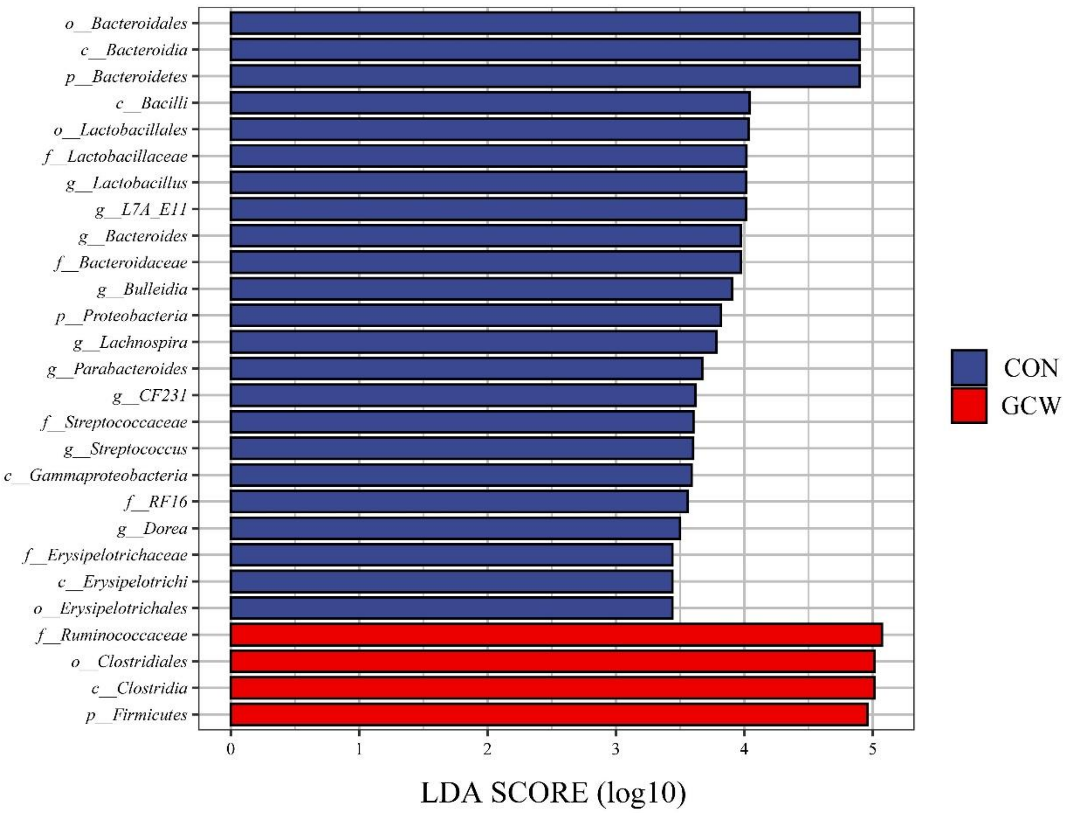

Supplementary Figure. S2. LEfSe analysis of gut microbiota between two groups. Blue shaded areas indicate CON-enriched taxa and red shaded areas indicate SF-enriched taxa.
